# Dietary Supplementation with *Zanthoxylum armatum* ‘Jiuyeqing’ Leaf Extract Affects Laying Performance, Antioxidant Status, and Hepatic Metabolomic Profiles in Laying Hens

**DOI:** 10.3390/vetsci13080729

**Published:** 2026-07-24

**Authors:** Qiaobo Lei, Juan Wang, Shanchuan Cao, Xiaocong Li, Jianfei Zhao, Jingbo Liu

**Affiliations:** 1 College of Life Sciences and Agri-Forestry, Southwest University of Science and Technology, Mianyang 621010, China; 2 Tongwei Agricultural Development Co., Ltd., Chengdu 610096, China

**Keywords:** phytogenic feed additive, egg quality, hepatic metabolome, glycerophospholipid metabolism

## Abstract

This study evaluated the effects of dietary *Zanthoxylum armatum* DC. ‘Jiuyeqing’ leaf extract (ZBLE) in laying hens. A total of 1024 healthy 41-week-old Lohmann Pink layers were assigned to four treatments with eight replicates of 32 hens and fed diets containing 0, 2, 4, or 6 g ZBLE/kg for 8 weeks. The results showed that laying rate significantly increased linearly during weeks 1–4 and 1–8 (*p* = 0.009 and 0.005, respectively), and the 6 g/kg group had a higher laying rate than the control group (CON) over weeks 1–8 (*p* = 0.026). Yolk color score increased (*p* < 0.001), and serum immunoglobulin M concentration increased significantly (*p* = 0.007), whereas serum biochemical parameters remained unchanged. Serum total antioxidant capacity and total superoxide dismutase activity and hepatic total antioxidant capacity and catalase activity increased significantly, whereas serum malondialdehyde decreased. Intestinal responses were segment-specific, with increased duodenal villus height, alongside greater jejunal crypt depth and a lower jejunal villus-to-crypt ratio at 6 g/kg. Hepatic metabolomic comparison between the CON and 6 g/kg groups indicated that glycerophospholipid metabolism was the most strongly enriched pathway. Overall, 6 g ZBLE/kg produced the most favorable response among the tested levels and may serve as a plant-based feed additive for laying hens.

## Simple Summary

Plant-based feed additives may support poultry production while providing new uses for agricultural plant resources. In this study, an extract prepared from the leaves of *Zanthoxylum armatum* DC. ‘Jiuyeqing’ was added to laying-hen diets for 8 weeks. Among the tested levels, 6 g/kg produced the best overall response, including higher egg production, deeper yolk color, stronger antioxidant protection, and changes in immune-related indicators. The extract also affected intestinal structure and liver metabolism, although the intestinal responses differed among regions. These findings suggest that *Z. armatum* leaves may be developed as a plant-based feed resource, providing a potential approach to support laying-hen production and increase the value of underused leaf materials.

## 1. Introduction

Eggs are widely consumed sources of high-quality protein, lipids, vitamins, and essential micronutrients. Laying hen production is a vital sector of the livestock industry. Maintaining a high and persistent laying rate is important for the profitability and sustainability of commercial layer production. However, laying hens are exposed to nutritional, environmental, microbial, and physiological challenges that may impair laying persistence and physiological homeostasis. Phytogenic feed additives have received increasing attention as non-antibiotic nutritional strategies in poultry production. Previous studies have shown that phytogenic feed additives can affect laying performance, egg quality, antioxidant status, immune-related responses, and intestinal function in laying hens [1,2,3,4]. However, these responses may vary according to plant source, extract preparation, inclusion level, and production conditions, emphasizing the need to evaluate each botanical additive under defined experimental conditions.

Phytochemical studies have shown that the leaves of *Zanthoxylum bungeanum* Maxim are rich in flavonoids, polyphenols, and essential oils and exhibit considerable free-radical-scavenging and antioxidant activities [5,6]. These activities have been associated primarily with the phenolic and flavonoid constituents of the leaves [7]. In laying hens, dietary inclusion of *Z. bungeanum* leaves has been reported to affect egg quality, antioxidant status, humoral immune-related variables, gut-related traits, yolk color, and yolk lipid composition without adversely affecting laying performance [8,9]. These findings indicate the potential nutritional value of *Zanthoxylum* leaves in poultry production.

Although these findings support the potential use of *Zanthoxylum* leaves in laying-hen nutrition, *Z. bungeanum* and *Z. armatum* are distinct species and may differ in their phytochemical characteristics [10]. *Z*. *armatum* DC. ‘Jiuyeqing’ is an important green prickly ash resource in southwestern China [11]. Its leaves contain phenolic and flavonoid compounds with antioxidant activity, although their composition may vary with plant age, leaf position, extraction conditions, and geographical origin [12,13]. These characteristics support the evaluation of ‘Jiuyeqing’ leaves as a botanical feed resource, but their effects in laying hens remain largely unknown.

Direct inclusion of raw leaves may introduce compositional variation, whereas extraction can enrich solvent-extractable phytochemicals and provide a more controlled preparation for dietary application [14,15]. Chemical characterization is therefore necessary for interpreting the biological effects of the extract [15]. The liver is central to egg formation because yolk precursors are synthesized and packaged there before transport to developing follicles [16]. Hepatic lipid synthesis, lipoprotein assembly, and membrane-lipid metabolism may therefore influence the supply of substrates required for sustained egg production [17].

Accordingly, the present study evaluated the effects of 0, 2, 4, and 6 g/kg *Z. armatum* DC. ‘Jiuyeqing’ leaf extract (ZBLE) on laying performance, egg quality, serum biochemical and humoral immune-related variables, antioxidant status, intestinal morphology, and hepatic metabolic profiles in laying hens. Hepatic metabolomics was conducted in the control and 6 g/kg groups to identify treatment-associated metabolic features.

## 2. Materials and Methods

### 2.1. Production of ZBLE

**Figure 1:**
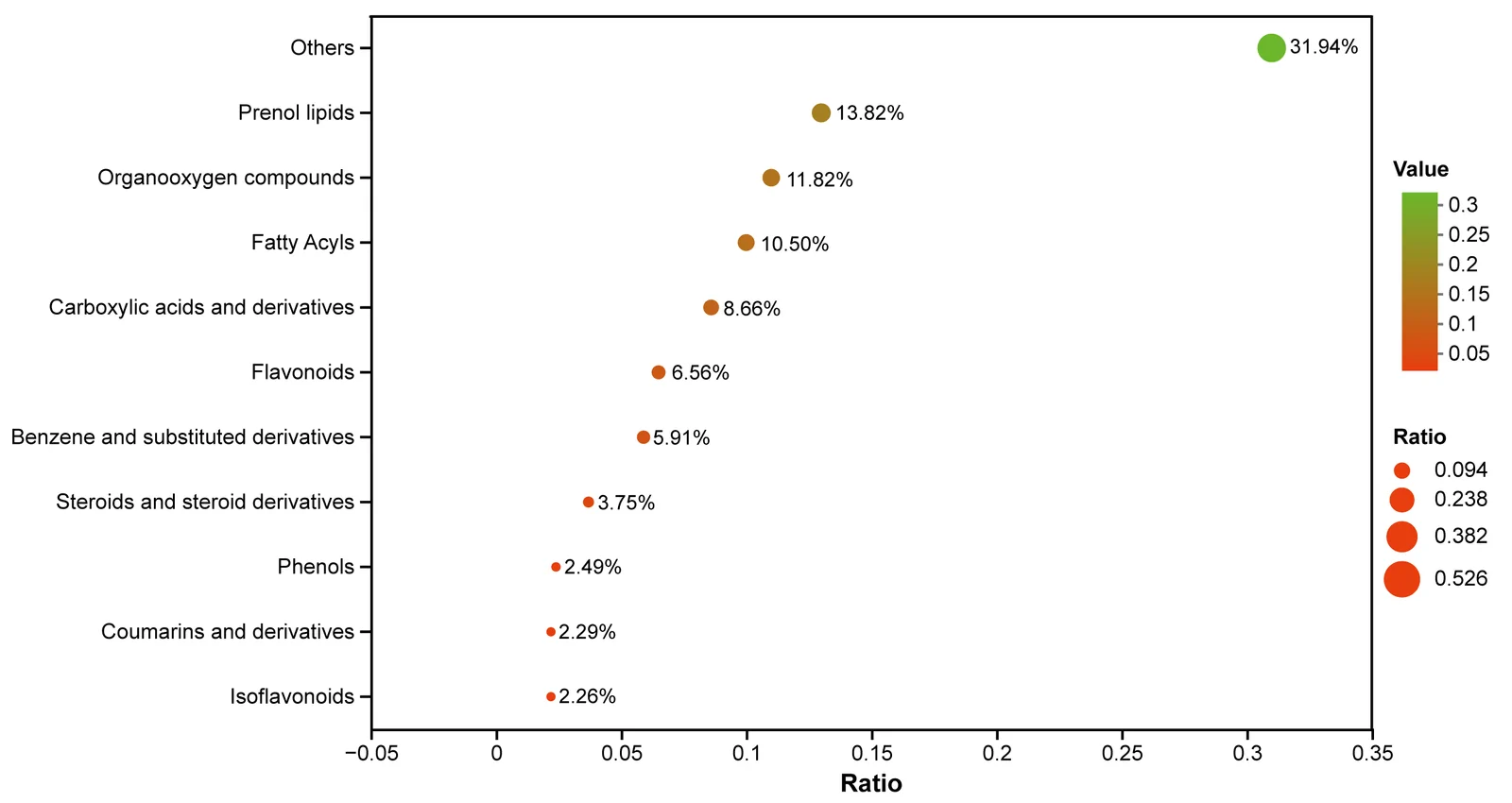
Relative distribution of annotated chemical classes in ZBLE based on normalized LC–MS peak areas. The percentages represent relative abundances.

Fresh leaves of *Z. armatum* DC. ‘Jiuyeqing’ were collected at commercial maturity in Nanchong, Sichuan Province, China. The leaves were rinsed with distilled water, surface-dried, dried in a forced-air oven at 45 °C to constant weight, and ground to pass through a 1-mm sieve. The dried leaf powder was stored in sealed containers at 4 °C and protected from light until extraction. The dried leaf powder was extracted three times with 70% aqueous ethanol (*v*/*v*) under reflux at 70 °C for 1 h, using a solid-to-liquid ratio of 1:25 (*w*/*v*). After each extraction, the mixture was filtered, and the three filtrates were pooled. The combined filtrate was concentrated under reduced pressure at 45 °C, dried at 40 °C to constant weight, ground, and passed through an 80-mesh sieve. The extraction procedure was conducted in five batches, and the resulting dried extracts were pooled and thoroughly homogenized. The extraction yield, calculated as the dry extract weight divided by the initial dry leaf powder weight × 100%, was 12.3%. The pooled crude extract was designated ZBLE and stored in airtight containers at −20 °C, protected from light, until diet preparation and chemical analysis. The total flavonoid content of ZBLE was determined using the sodium nitrite–aluminum nitrate–sodium hydroxide colorimetric method. Briefly, the sample solution was reacted sequentially with sodium nitrite, aluminum nitrate, and sodium hydroxide, and the absorbance was measured at 510 nm. Rutin was used to construct the calibration curve, and the results were expressed as rutin equivalents (RE). The total phenolic content was determined using the Folin–Ciocalteu colorimetric method. After reaction with Folin–Ciocalteu reagent and sodium carbonate, the absorbance was measured at 765 nm. Gallic acid was used to construct the calibration curve, and the results were expressed as gallic acid equivalents (GAE). The total flavonoid and total phenolic contents of ZBLE were 266.5 g RE/kg extract and 216.8 g GAE/kg extract, respectively. Untargeted LC–MS profiling further showed that flavonoids, isoflavonoids, and phenols accounted for 6.56%, 2.26%, and 2.49% of the normalized LC–MS peak area, respectively (Figure 1). These percentages represent the relative proportions of the corresponding annotated chemical classes.

### 2.2. Experimental Design, Diets, and Management

A total of 1024 healthy 41-week-old Lohmann Pink laying hens with comparable initial body weights and laying rates were allocated to four dietary treatments in a completely randomized design. Each treatment consisted of eight replicates, with 32 hens per replicate. Each replicate comprised 16 adjacent cages within the same row, with two hens housed per cage, and the replicate was considered the experimental unit. The four dietary treatments contained 0, 2, 4, and 6 g ZBLE/kg diet, respectively. The control diet contained no ZBLE. The basal diet was formulated to meet the nutritional requirements of laying hens. ZBLE was incorporated into the experimental diets by replacing corn on an equal-weight basis, while the inclusion levels of the other dietary ingredients remained unchanged. Accordingly, the corn inclusion levels were 607, 605, 603, and 601 g/kg diet in the groups receiving 0, 2, 4, and 6 g ZBLE/kg diet, respectively. The ingredient composition and nutrient levels of the experimental diets are presented in Table 1. The basal diet was formulated according to the NRC (1994) [18] and the Chinese Feeding Standard of Chicken (NY/T 33-2004) [19]. All experimental diets were prepared as mash feed and thoroughly mixed at a commercial feed mill to ensure the homogeneous distribution of ZBLE. The hens were provided with a 7-day adaptation period, followed by an 8-week feeding trial. Birds were housed in an open-sided, naturally ventilated laying house equipped with four-tier stainless-steel cages measuring 70 × 50 × 60 cm. Feed intake was measured at the replicate level. The amounts of feed supplied and residual feed from all cages within each replicate were recorded weekly and used to calculate feed consumption for each replicate. Feed and fresh water were provided ad libitum throughout the trial. The ambient temperature was maintained at 20–22 °C, and the relative humidity ranged from 40% to 60%. A photoperiod of 16 h light and 8 h dark was applied daily. The health status and mortality of the hens were monitored and recorded at least once daily.

### 2.3. Laying Performance and Sample Collection

Laying performance was recorded for each replicate throughout the 8-week feeding trial. The number and total weight of eggs produced were recorded daily for each replicate. Hen-day laying rate was calculated as the total number of eggs divided by the total number of hen-days × 100%. Average egg weight was determined by dividing the total egg mass by the total number of eggs. The amounts of feed offered and residual feed were recorded weekly for each replicate, and feed consumption was calculated as the difference between these values. Average daily feed intake (ADFI) was calculated as the total feed consumed divided by the total number of hen-days. Daily egg mass was calculated as hen-day laying rate × average egg weight/100 and was expressed as g/hen/day. Feed conversion ratio (FCR) was calculated as total feed intake divided by total egg mass.

At the conclusion of the 8-week feeding trial, only clinically healthy hens without visible injuries or anatomical abnormalities were included in the sampling pool. One hen was randomly selected from each replicate, resulting in eight independent biological replicates per treatment. Before sampling, feed was withdrawn for 12 h, whereas water remained available ad libitum. After live body weight was recorded, blood samples were collected from the wing vein into vacuum blood-collection tubes. The blood was allowed to clot at room temperature and then centrifuged at 3000× *g* for 10 min. The serum was collected, aliquoted, and stored at −80 °C for later testing.

All selected hens were humanely slaughtered, and the liver, spleen, ovary and oviduct were dissected and weighed. The liver, spleen, ovarian, and oviduct indices were calculated individually as organ weight (g)/live body weight (g) × 100 and were expressed as percentages of live body weight.

Intestinal segments of approximately 2 cm were collected from the duodenum, jejunum and ileum; gently rinsed with phosphate-buffered saline (PBS); and fixed in 4% formaldehyde for hematoxylin and eosin (H&E) staining and intestinal morphometric analysis. Liver tissue samples were divided into aliquots, immediately frozen in liquid nitrogen, and stored at −80 °C until biochemical and metabolomic analyses.

### 2.4. Egg Quality

Egg quality was assessed at the end of the 8-week feeding trial. On the final day of the experiment, four eggs were randomly selected from each replicate, resulting in 32 eggs per treatment. Surface contaminants were gently removed, and each egg was weighed using an analytical balance (FA1604N, Shanghai Precision Scientific Instruments Co., Shanghai, China). Egg length and width were measured using a digital vernier caliper. The egg length-to-width ratio was calculated by dividing egg length by egg width. The mean value of the four eggs collected from each replicate was used as the replicate value for statistical analysis.

Eggshell color was measured at the equatorial region using a CR-400 colorimeter (Konica Minolta, Tokyo, Japan). The L, a, and b* values were recorded at three equidistant points around the equator of each egg, and the three measurements were averaged.

After each egg was broken onto the measurement platform, albumen height and yolk color score were measured using an Egg Multi Tester (EMT-5200, Robotmation Co., Tokyo, Japan). The Haugh unit was determined automatically by the instrument based on egg weight and albumen height. The eggshell force gauge (Model II, Robotmation Co., Tokyo, Japan) was used to determine eggshell breaking strength at the equatorial region. Following removal of the egg contents and shell membranes, the eggshells were thoroughly washed, dried at 65 °C, and weighed. Eggshell thickness was measured at the blunt end, pointed end, and equator using a digital micrometer, and the mean of the three measurements was recorded for each egg.

### 2.5. Intestinal Morphology

Duodenal, jejunal, and ileal samples fixed in 4% formaldehyde were processed and evaluated separately. The samples were dehydrated through a graded ethanol series, cleared in xylene, embedded in paraffin, sectioned, and stained with H&E. The stained sections were examined and photographed at 40× magnification using a digital optical microscope equipped with a BA400 digital camera system (McAudi Industrial Group Co., Ltd., Xiamen, China). For each intestinal segment from each hen, 10 intact and well-oriented villus–crypt units were randomly selected for measurement on calibrated digital images. Villus height was measured from the tip of the villus to the villus–crypt junction, whereas crypt depth was measured from the base of the crypt to the villus–crypt junction. The villus height-to-crypt depth ratio (V/C) was calculated by dividing villus height by crypt depth. The mean of the 10 measurements obtained from each intestinal segment of each hen was used as the replicate value for statistical analysis, resulting in eight biological replicates per treatment.

### 2.6. Serum Biochemistry and Immunoglobulins

Serum biochemical parameters were analyzed using an automated biochemical analyzer (Hitachi 7020, Hitachi, Tokyo, Japan). The parameters measured were alkaline phosphatase (ALP), aspartate aminotransferase (AST), urea, alanine aminotransferase (ALT), albumin (ALB), cholesterol (CHO), triglycerides (TG), and total protein (TP). ALP activity was determined using a p-nitrophenyl phosphate kinetic method, whereas ALT and AST activities were determined using kinetic enzymatic methods. Serum urea was measured using a urease-based enzymatic method, ALB using the bromocresol green method, TP using the biuret method, CHO using the cholesterol oxidase–peroxidase method, and TG using the glycerol phosphate oxidase–peroxidase method. All measurements were performed according to the corresponding reagent instructions and the operating procedures of the automated analyzer. The concentrations of immunoglobulin A (IgA), immunoglobulin M (IgM), and immunoglobulin G (IgG) were quantified using commercial ELISA kits (Nanjing Jiancheng Bioengineering Institute, Nanjing, China) according to the manufacturer’s instructions. The assays were based on specific antigen–antibody binding and enzyme-mediated color development. The serum concentrations of IgA, IgM, and IgG were calculated from the corresponding standard curves.

### 2.7. Antioxidant Capacity

Antioxidant status in serum and liver tissue was evaluated by measuring total antioxidant capacity (T-AOC), catalase (CAT) activity, malondialdehyde (MDA) levels, and total superoxide dismutase (T-SOD) activity. Liver tissue was homogenized under ice-cold conditions and centrifuged, and the resulting supernatant was used for analysis. All indicators were measured using commercial assay kits (Nanjing Jiancheng Bioengineering Institute, Nanjing, China) according to the manufacturer’s instructions. T-AOC was determined using a colorimetric method. CAT activity was measured using the ammonium molybdate method based on the enzymatic decomposition of hydrogen peroxide. MDA levels were determined using the thiobarbituric acid method, whereas T-SOD activity was measured using the hydroxylamine method based on the inhibition of superoxide-mediated hydroxylamine oxidation. Serum results were expressed as U/mL or nmol/mL, whereas liver results were normalized to the protein concentration of the tissue homogenate and expressed as U/mg protein or nmol/mg protein. These indicators were selected to provide complementary information on overall antioxidant capacity, enzymatic defense against superoxide radicals and hydrogen peroxide, and the extent of lipid peroxidation.

### 2.8. Untargeted LC–MS Profiling of ZBLE and Hepatic Metabolomics

Untargeted liquid chromatography–mass spectrometry (LC–MS) analysis was performed by Shanghai Majorbio Bio-pharm Technology Co., Ltd. (Shanghai, China). To compare hepatic metabolic profiles between the control and highest ZBLE supplementation levels, liver samples from the CON and 6 g/kg ZBLE groups were subjected to untargeted metabolomic analysis. Eight liver samples were initially collected from each group. Following sample preparation and quality assessment, six samples per group met the analytical quality requirements and were included in the final analysis, resulting in six biological replicates per group. A pooled and homogenized ZBLE sample prepared as described in Section 2.1 was analyzed in parallel using the same LC–MS platform. For metabolite extraction, 100 mg of each liver or ZBLE sample was transferred into a 2 mL centrifuge tube containing a 6 mm grinding bead. Subsequently, 800 μL of methanol/water extraction solution (4:1, *v*/*v*) containing an internal-standard mixture, including L-2-chlorophenylalanine at 0.02 mg/mL, was added. The samples were homogenized at −10 °C for 6 min at 50 Hz, ultrasonicated at 5 °C for 30 min at 40 kHz, maintained at −20 °C for 30 min, and centrifuged at 13,000× *g* for 15 min at 4 °C. The resulting supernatants were transferred into autosampler vials with inserts for LC–MS analysis. Quality control (QC) samples were prepared by pooling equal aliquots of all liver-sample extracts and were injected periodically throughout the analytical sequence to monitor instrument stability and analytical reproducibility. Metabolite separation was performed using an ultra-high-performance liquid chromatography system equipped with an HSS T3 column (100 mm × 2.1 mm, 1.8 μm). Mobile phase A consisted of water/acetonitrile (95:5, *v*/*v*) containing 0.1% formic acid, whereas mobile phase B consisted of acetonitrile/isopropanol/water (47.5:47.5:5, *v*/*v*/*v*) containing 0.1% formic acid. The flow rate was maintained at 0.40 mL/min, and the column temperature was set at 40 °C. Mass-spectrometric data were acquired separately in positive- and negative-ion modes over a scan range of *m*/*z* 70–1050. The spray voltage was set at 3500 V in positive-ion mode and −3000 V in negative-ion mode. The sheath-gas and auxiliary-gas flow rates were set at 50 and 13 arbitrary units, respectively. The ion-source temperature was maintained at 450 °C, and stepped collision energies of 20, 40, and 60 V were applied for MS/MS acquisition. Raw LC–MS data were processed using Progenesis QI software, version 3.1 (Waters Corporation, Milford, MA, USA) for baseline correction, peak detection, peak integration, retention-time correction, alignment, and normalization. Metabolites were annotated by matching accurate mass, isotope distribution, and MS/MS fragmentation patterns against public databases, including the Human Metabolome Database (HMDB) and METLIN, as well as the Majorbio in-house database. Positive- and negative-ion datasets were initially processed and annotated separately. The resulting annotated metabolite tables were subsequently combined, and duplicate metabolite annotations were removed. Metabolic features detected in at least 80% of the samples in either treatment group were retained. Remaining missing values were replaced with the minimum value observed in the data matrix. Features with a relative standard deviation greater than 30% in the QC samples were excluded because of insufficient analytical reproducibility. The filtered peak-intensity data were normalized and log10-transformed before subsequent statistical analysis. Principal component analysis (PCA) was conducted to evaluate overall metabolic variation between groups. Orthogonal partial least-squares discriminant analysis (OPLS-DA) was performed to identify metabolites contributing to group separation. The OPLS-DA model was evaluated using R^2^Y and Q^2^ values, and model overfitting was assessed using 200 permutation tests. Differential metabolites were screened using a variable importance in projection (VIP) value greater than 1.0 and a Benjamini–Hochberg false-discovery-rate-adjusted *p* value below 0.05. Differential metabolites were annotated and subjected to pathway enrichment analysis using the Kyoto Encyclopedia of Genes and Genomes (KEGG) database.

### 2.9. Statistical Analysis

The data were analyzed using SAS software, version 9.3 (SAS Institute Inc., Cary, NC, USA). The replicate was considered the experimental unit for all analyses. Laying-performance variables were calculated for each replicate. For egg-quality measurements, the mean of the four eggs collected from each replicate was used as the replicate value. For serum, organ-index, antioxidant-status, and intestinal-morphology variables, the value obtained from the hen sampled from each replicate was used for statistical analysis. Data were analyzed using one-way analysis of variance (ANOVA) with the general linear model procedure. Linear and quadratic responses to increasing dietary ZBLE supplementation were evaluated using orthogonal polynomial contrasts. Because the four supplementation levels were equally spaced at 0, 2, 4, and 6 g/kg diet, the contrast coefficients were −3, −1, 1, and 3 for the linear response and 1, −1, −1, and 1 for the quadratic response, respectively. When the overall F-test showed statistical significance, Tukey’s Honestly Significant Difference (HSD) test was used to compare differences among group means. Data were expressed as least-squares means with the pooled standard error of the mean (SEM). Differences were considered statistically significant at *p* < 0.05, whereas 0.05 ≤ *p* < 0.10 was interpreted as a tendency.

## 3. Results

### 3.1. Laying Performance and Egg Quality

As shown in Table 2, laying rate increased linearly with increasing dietary ZBLE supplementation during weeks 1–4 and throughout weeks 1–8 (linear *p* = 0.009 and 0.005, respectively). The overall treatment effect during weeks 1–4 showed a tendency (ANOVA *p* = 0.063), whereas the effect across weeks 1–8 was significant (ANOVA *p* = 0.026), with a higher laying rate in the 6 g/kg ZBLE group than in the CON group (*p* < 0.05). Dietary ZBLE supplementation did not significantly affect laying rate during weeks 5–8, ADFI, average egg weight, or feed conversion ratio (*p* > 0.05).

As shown in Table 3, yolk color score increased linearly with increasing ZBLE supplementation (linear *p* < 0.001). The scores in the 4 and 6 g/kg ZBLE groups were higher than that in the CON group, and the score in the 6 g/kg group was also higher than that in the 2 g/kg group (*p* < 0.05). Eggshell L* showed a quadratic response (quadratic *p* = 0.041), while albumen height and the Haugh unit showed linear increasing trends (linear *p* = 0.058 and 0.080, respectively).

### 3.2. Serum Biochemistry and Immunoglobulins

As shown in Table 4, dietary ZBLE supplementation had no significant effects on serum biochemical parameters (*p* > 0.05). However, serum IgM concentration increased from 276.75 in the CON group to 380.56 in the 6 g/kg ZBLE group, and the value in the 6 g/kg group was significantly higher than those in the CON and 2 g/kg groups (ANOVA *p* = 0.007). Furthermore, serum IgM and IgA concentrations increased linearly with increasing dietary ZBLE supplementation (linear *p* < 0.001 and *p* = 0.036, respectively).

### 3.3. Organ Indices and Antioxidant Capacity

Dietary ZBLE supplementation did not significantly affect body weight, absolute organ weights, or organ indices (Table 5, *p* > 0.05). As shown in Table 6, serum T-AOC increased from 4.23 U/mL in the CON group to 6.18–6.59 U/mL in the ZBLE-supplemented groups (ANOVA *p* = 0.009; linear *p* = 0.007; quadratic *p* = 0.028). Serum MDA decreased from 5.35 nmol/mL in the CON group to 2.99 nmol/mL in the 6 g/kg group (ANOVA *p* = 0.019; linear *p* = 0.002), whereas serum T-SOD increased from 107.56 to 143.53 U/mL (ANOVA *p* = 0.004). Hepatic T-AOC increased from 4.15 U/mg protein in the CON group to 6.32 U/mg protein in the 6 g/kg group (ANOVA *p* = 0.020; linear *p* = 0.003). Hepatic CAT activity was 8.48 U/mg protein in the CON group and 12.35 U/mg protein in the 6 g/kg group, with a significant linear response (*p* = 0.003). Hepatic MDA decreased from 1.73 nmol/mg protein in the CON group to its lowest value of 0.96 nmol/mg protein in the 4 g/kg group and subsequently increased to 1.44 nmol/mg protein in the 6 g/kg group (ANOVA *p* = 0.027; quadratic *p* = 0.017). Serum CAT and hepatic T-SOD were not significantly affected (ANOVA *p* = 0.615 and 0.496, respectively).

### 3.4. Intestinal Morphology

As shown in Table 7, dietary ZBLE supplementation increased duodenal villus height, with higher values observed in the 4 and 6 g/kg groups than in the CON and 2 g/kg groups (ANOVA *p* < 0.001; linear *p* < 0.001). The duodenal V/C ratio also increased linearly with increasing ZBLE supplementation (linear *p* = 0.010), although the overall treatment effect showed only a tendency (ANOVA *p* = 0.061). In the jejunum, crypt depth showed a significant quadratic response to ZBLE supplementation (ANOVA *p* = 0.017; quadratic *p* = 0.012), with a higher value in the 6 g/kg group than in the 2 g/kg group. The jejunal V/C ratio decreased at the highest supplementation level and showed significant linear and quadratic responses (ANOVA *p* = 0.009; linear *p* = 0.015; quadratic *p* = 0.013). Ileal villus height and V/C ratio showed decreasing linear trends (linear *p* = 0.062 and 0.059, respectively), whereas no significant effects were observed for the remaining ileal morphological variables.

### 3.5. Hepatic Metabolome

**Figure 2:**
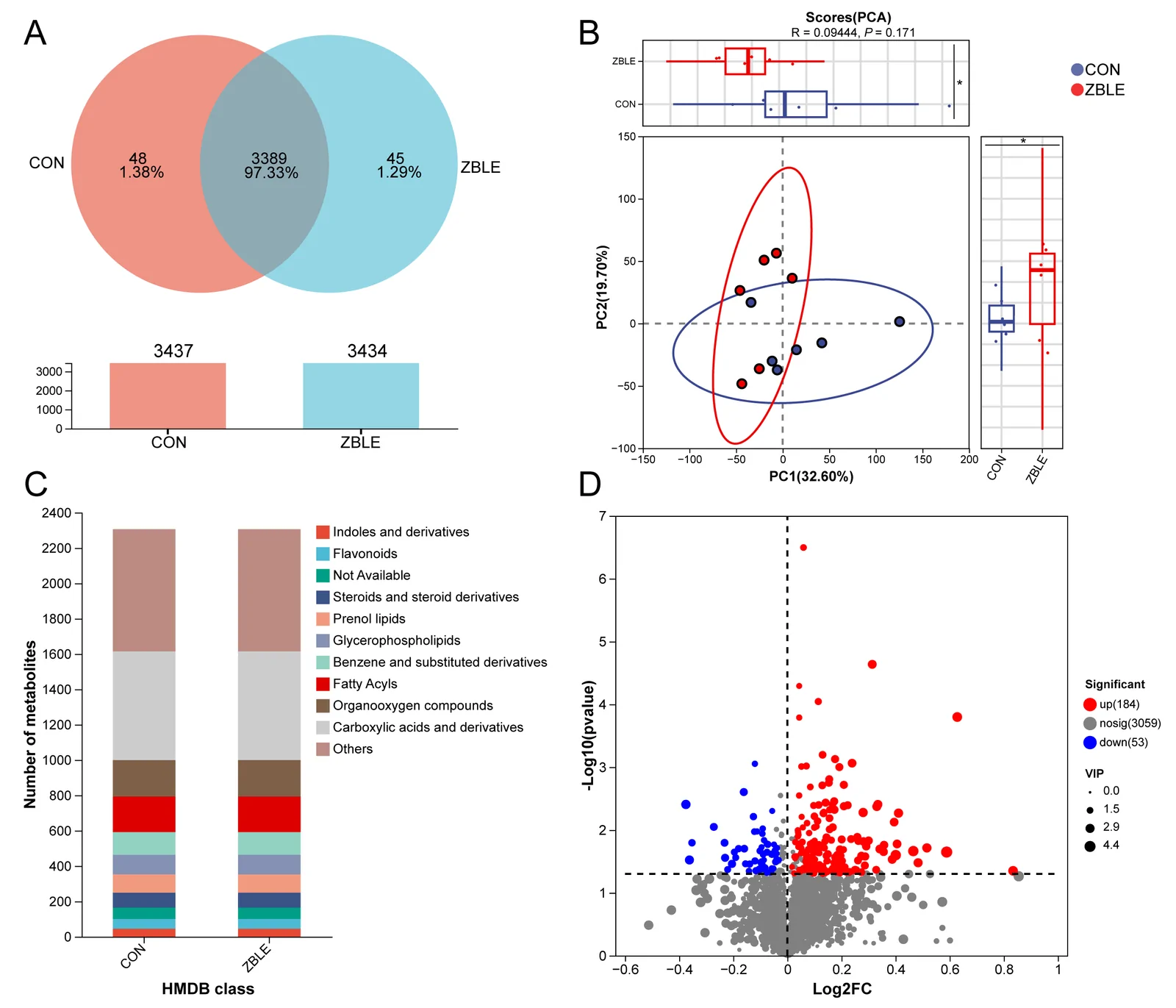
Overview of hepatic metabolomic profiles in the CON and 6 g/kg ZBLE groups. (**A**) Venn diagram showing the numbers of shared and uniquely detected annotated metabolites in the two groups; the bar chart shows the total number of annotated metabolites detected in each group. (**B**) Principal component analysis score plot of hepatic metabolomic profiles. Each point represents one biological replicate, and the ellipses indicate the distribution of samples within each group. The first and second principal components explained 32.60% and 19.70% of the total variation, respectively. (**C**) Distribution of annotated hepatic metabolites according to Human Metabolome Database chemical classes. (**D**) Volcano plot of differential metabolites between the 6 g/kg ZBLE and CON groups. Red and blue points represent metabolites with higher and lower abundance, respectively, in the 6 g/kg ZBLE group, whereas gray points represent metabolites that did not meet the differential-screening criteria. Point size represents the variable importance in projection value. CON, control diet; ZBLE, *Zanthoxylum armatum* DC. ‘Jiuyeqing’ leaf extract; PCA, principal component analysis; VIP, variable importance in projection. *n* = 6 biological replicates per group. * *p* < 0.05.

**Figure 3:**
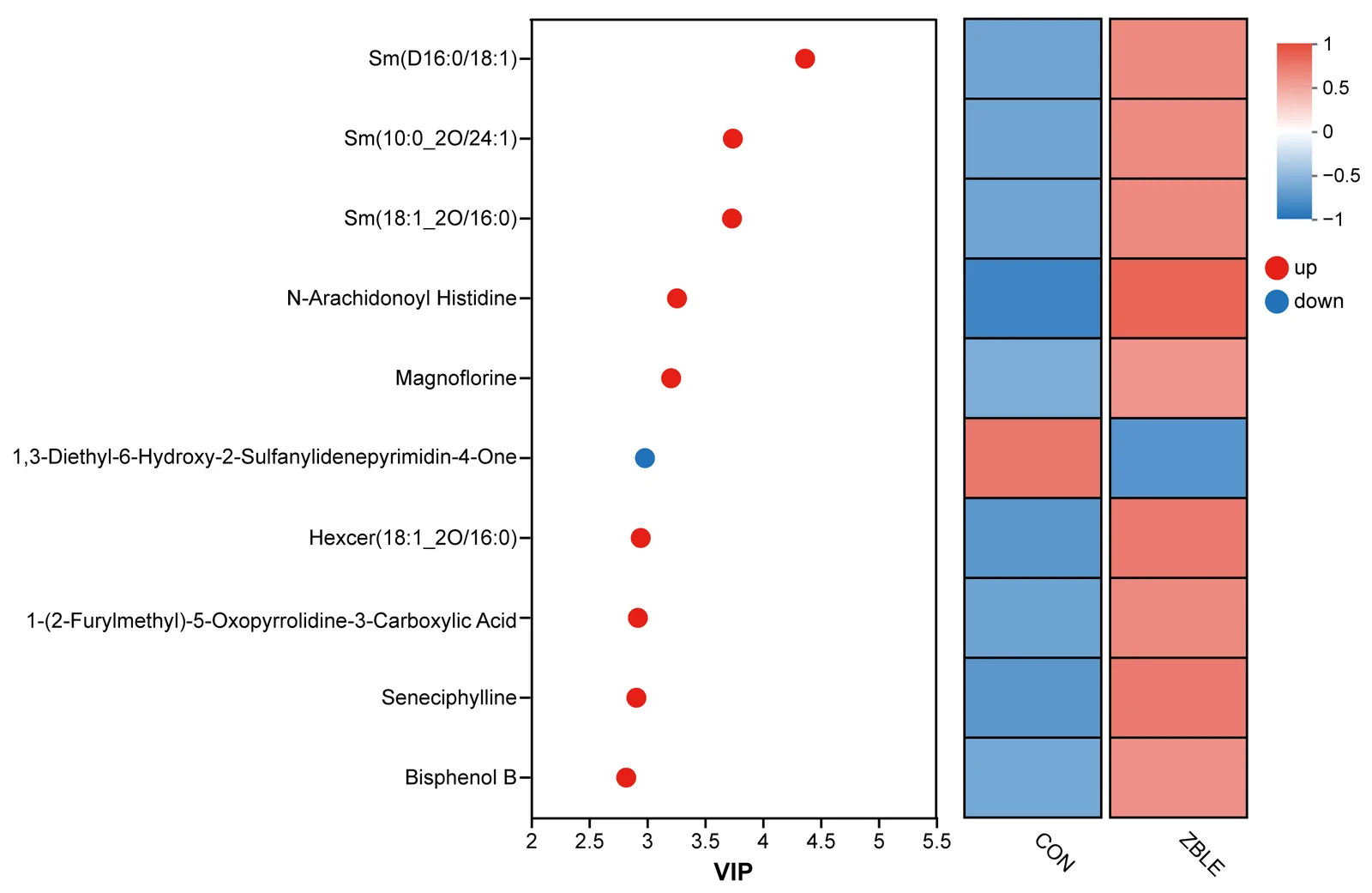
Variable importance in projection scores and relative abundance patterns of the top 10 differential hepatic metabolites between the CON and 6 g/kg ZBLE groups. The left panel shows the top 10 differential metabolites ranked according to their variable importance in projection (VIP) values derived from the OPLS-DA model. Differential metabolites were screened using VIP > 1.0 and a Benjamini–Hochberg FDR-adjusted *p* value < 0.05. Red and blue points indicate metabolites with higher and lower abundance, respectively, in the ZBLE group relative to the CON group. The right panel shows the standardized relative abundance of each metabolite in the two groups, with red and blue representing higher and lower relative abundance, respectively. CON, control diet; ZBLE, *Zanthoxylum armatum* DC. ‘Jiuyeqing’ leaf extract; VIP, variable importance in projection; OPLS-DA, orthogonal partial least-squares discriminant analysis. *n* = 6 biological replicates per group.

**Figure 4:**
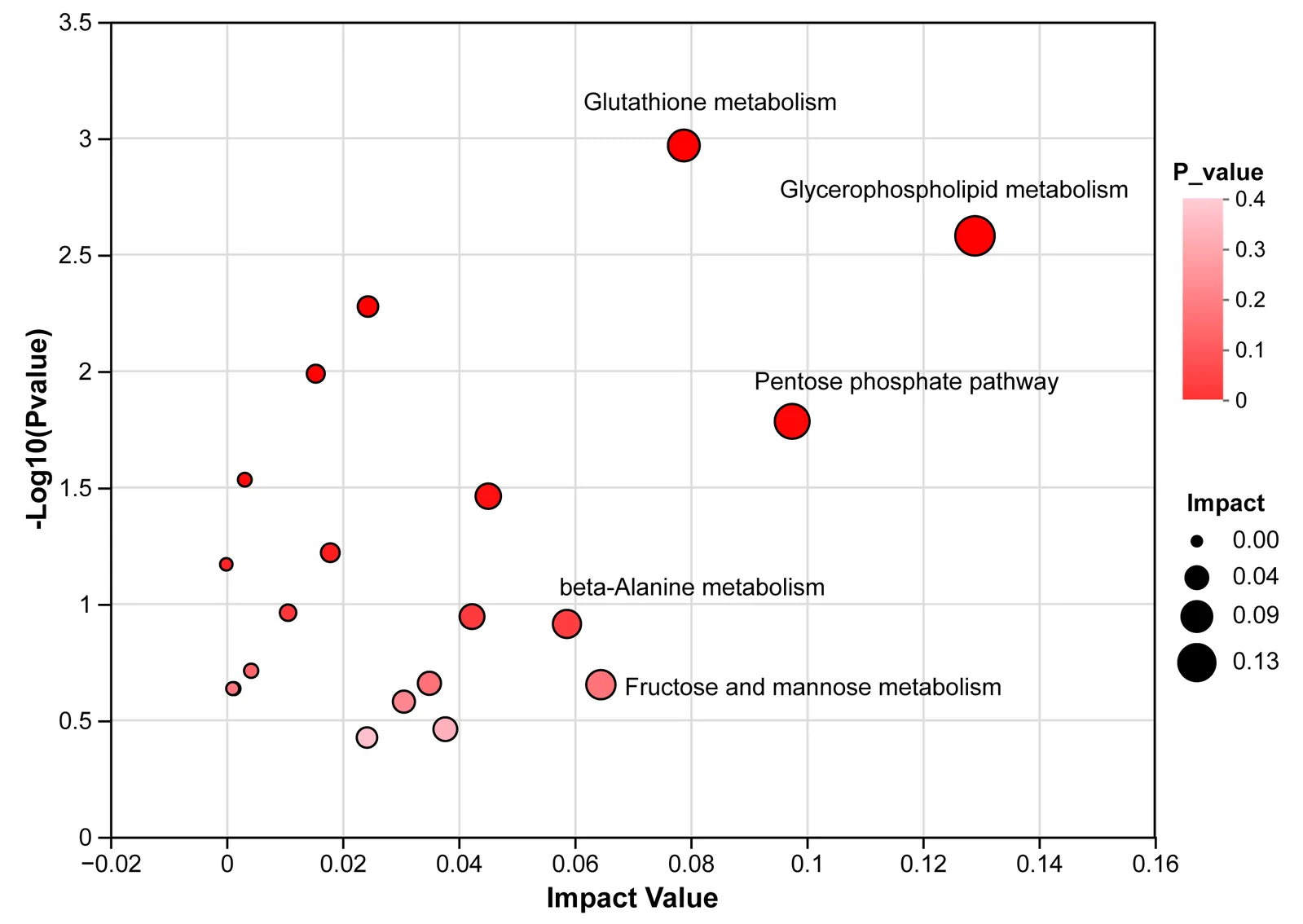
KEGG pathway enrichment and topology analysis of differential hepatic metabolites between the CON and 6 g/kg ZBLE groups. Each bubble represents a metabolic pathway. The horizontal axis indicates the pathway impact value derived from topology analysis, whereas the vertical axis represents −log10(*p* value) from pathway enrichment analysis. Bubble size is proportional to the pathway impact value, and bubble color indicates the enrichment *p* value, with darker red representing a lower *p* value. Labeled pathways include glutathione metabolism, glycerophospholipid metabolism, the pentose phosphate pathway, β-alanine metabolism, and fructose and mannose metabolism. CON, control diet; ZBLE, *Zanthoxylum armatum* DC. ‘Jiuyeqing’ leaf extract.

As shown in Figure 2A, 3389 annotated metabolites were shared between the CON and 6 g/kg ZBLE groups, whereas 48 and 45 metabolites were uniquely detected in the CON and ZBLE groups, respectively. The first two principal components explained 32.60% and 19.70% of the total variation, respectively; however, the overall separation between the two groups was not statistically significant (R = 0.094, *p* = 0.171; Figure 2B). The two groups showed broadly similar distributions of HMDB chemical classes, with carboxylic acids and derivatives, organooxygen compounds, and fatty acyls among the predominant annotated classes (Figure 2C). A total of 237 differential metabolites were identified between the two groups, of which 184 were more abundant and 53 were less abundant in the 6 g/kg ZBLE group than in the CON group (Figure 2D). SM(d16:0/18:1) had the highest VIP score among the differential metabolites (Figure 3). KEGG pathway analysis showed that glycerophospholipid metabolism exhibited the strongest enrichment signal (Figure 4).

## 4. Discussion

Laying performance is a major determinant of the economic efficiency of commercial egg production. Previous studies have shown that adding *Yucca schidigera* extract to the diet improves feed conversion ratio and egg weight [21]. *Allium* extract increases egg weight and egg size [22]. Mulberry leaf extract has no adverse effects on egg production performance [23]. Although the addition of grape extract reduces egg weight, it does not affect the egg production rate [24]. These contrasting responses indicate that the effects of phytogenic additives on laying performance depend on their botanical origin, chemical composition, extraction procedure, and inclusion level. In the present study, laying rate increased linearly during weeks 1–4 and throughout weeks 1–8, and hens receiving 6 g/kg ZBLE had a higher laying rate than the control hens over the entire experimental period. Similar improvements in laying performance have been reported following supplementation with antioxidant-rich botanical preparations [3,25]. Phenolic and flavonoid constituents may support productive processes by maintaining cellular redox balance and nutrient metabolism. Egg quality is also an important determinant of consumer acceptance. Most egg-quality traits were not significantly altered by ZBLE supplementation, whereas yolk color increased significantly with increasing dietary ZBLE. Studies have shown that adding ingredients containing carotenoids and lutein to the diet can enhance the color of egg yolks [26]. Dietary inclusion of *Z. bungeanum* leaves has similarly been reported to deepen yolk color and alter yolk lipid composition [9]. Among the tested supplementation levels, 6 g/kg ZBLE produced the most favorable combination of laying-rate and yolk-color responses.

Serum levels of ALT and AST are commonly used as indicators of hepatocellular injury. In the event of liver cell damage or disruption of cell-membrane integrity, the release of ALT and AST into the serum results in elevated levels [27]. Dietary ZBLE did not significantly affect the measured serum biochemical variables or organ indices, indicating that no overt treatment-related abnormalities in these indicators occurred under the present experimental conditions. Serum IgM concentration increased significantly, whereas IgA showed a positive linear response to increasing ZBLE supplementation. These two immunoglobulins are essential components of mucosal and systemic humoral immunity in chickens. Elevated circulating IgA and IgM levels generally reflect enhanced immune competence in birds, regardless of normal or stressed conditions [28]. The increase in IgM may therefore reflect enhanced humoral immune responsiveness. Plant-derived polyphenols have been reported to influence humoral immunity through their effects on immune-cell activity, oxidative balance, and the intestinal environment [29,30]. In addition, we observed an increase in total antioxidant capacity in serum and liver tissue. Dietary ZBLE significantly increased serum T-AOC and T-SOD activities and hepatic T-AOC and CAT activities, whereas serum MDA concentration decreased significantly. Hepatic MDA showed a quadratic response and reached its lowest value at 4 g/kg. T-AOC reflects the combined contribution of enzymatic and non-enzymatic antioxidant components and is therefore commonly used as an integrated indicator of antioxidant capacity [31]. MDA is a major product of lipid peroxidation and is widely used to evaluate oxidative damage to membrane lipids [32]. The simultaneous increases in antioxidant capacity and enzyme activities, together with the reduction in MDA, indicate that ZBLE strengthened antioxidant defense and reduced lipid peroxidation in laying hens.

Flavonoids and phenolic compounds can contribute to antioxidant defense through radical scavenging, metal-ion chelation, and regulation of redox-sensitive cellular processes [33,34]. Chemical characterization showed that ZBLE contained 266.5 g rutin equivalents/kg extract of total flavonoids and 216.8 g gallic acid equivalents/kg extract of total phenolics. LC–MS annotation further showed that flavonoids, isoflavonoids, and phenols accounted for 6.56%, 2.26%, and 2.49% of the normalized peak area, respectively. The spectrophotometric measurements represent total flavonoid and phenolic equivalents, whereas the LC–MS results represent relative signal proportions of annotated chemical classes. Therefore, the two sets of values describe different analytical characteristics of the extract. Collectively, the phenolic and flavonoid profile of ZBLE provides a plausible phytochemical basis for the observed antioxidant and humoral immune-related responses.

Histological examination of the small intestine did not reveal obvious inflammatory lesions, but the morphological responses to ZBLE differed among intestinal segments. Increasing the height of the villi increases the intestinal surface area and may favor nutrient absorption [35,36]. ZBLE increased duodenal villus height, suggesting an increase in the potential absorptive surface of this segment. In contrast, hens receiving 6 g/kg ZBLE had greater jejunal crypt depth and a lower jejunal villus-to-crypt ratio, while ileal villus height and the villus-to-crypt ratio showed decreasing trends. Increased crypt depth may reflect enhanced epithelial-cell proliferation and turnover, whereas a lower villus-to-crypt ratio may indicate that crypt-cell renewal and villus maturation were not proportionally coordinated. Regional differences in digestive activity, luminal exposure, epithelial turnover, and microbial density may contribute to these segment-specific responses [37].

The liver is the primary organ responsible for the metabolism of nutrients such as carbohydrates, proteins, and fats. Among the tested supplementation levels, 6 g/kg ZBLE produced the most favorable overall laying and antioxidant responses. Hepatic metabolomic analysis was therefore conducted to compare this treatment with the control group and to identify associated metabolic features. Because only these two groups were compared, the metabolomic results characterize treatment-associated differences rather than a dose–response relationship. Although the overall PCA profiles of the two groups were not significantly separated, differential-metabolite and pathway analyses identified specific treatment-associated changes in hepatic metabolism. Topological analysis revealed that glycerophospholipid metabolism had the highest pathway-impact value following ZBLE supplementation. Glycerophospholipids are major structural components of cellular and organelle membranes and participate in membrane remodeling, cellular signaling, and lipoprotein assembly. In laying hens, phosphatidylcholine and related phospholipids are essential for lipoprotein structure and lipid transport [38]. Recent poultry studies further support the importance of VLDL assembly and secretion, yolk precursor formation, and hepatic lipid metabolism in follicular development and egg-yolk lipid deposition [39,40]. The enrichment of glycerophospholipid metabolism may therefore reflect changes in hepatic membrane-lipid remodeling and lipid-packaging processes associated with egg formation. Previous studies have shown that phytogenic compounds can influence hepatic lipid handling and membrane-lipid composition in poultry [41,42], while VLDL-related lipoprotein assembly is an important component of avian lipid transport [39]. However, serum TG and TC concentrations were unchanged in the present study, suggesting that the hepatic metabolic response was more closely related to lipid remodeling and packaging than to a generalized reduction in circulating lipid concentrations. This interpretation is also consistent with the role of the laying-hen liver in supplying lipid-rich yolk precursors rather than primarily depositing lipids in peripheral tissues.

In addition, we found that SM(d16:0/18:1) had the highest VIP value among the differential metabolites. This metabolite is a sphingomyelin species involved in membrane organization and sphingolipid signaling. Sphingomyelin plays a crucial role in cellular signaling and in regulating lipid metabolism and transport. Sphingomyelin metabolism generates bioactive lipids, including ceramide, sphingosine, and sphingosine-1-phosphate, which participate in membrane turnover, cellular stress responses, and lipid-related signaling [43]. In addition, sphingolipids serve as pivotal regulators of cell proliferation, differentiation, and programmed cell death throughout ovarian folliculogenesis [44]. The change in SM(d16:0/18:1) therefore suggests that ZBLE supplementation was associated with remodeling of hepatic membrane lipids and sphingolipid-related metabolic processes.

Taken together, the present findings indicate that dietary supplementation with *Z. armatum* DC. ‘Jiuyeqing’ leaf extract influenced laying performance, yolk color, humoral immune-related variables, antioxidant status, segment-specific intestinal morphology, and hepatic lipid-related metabolism. The study also demonstrates the potential value of an underused leaf resource as a phytogenic feed ingredient for laying hens. Several limitations should nevertheless be acknowledged. The feeding period was limited to 8 weeks, and hepatic metabolomics compared only the control and 6 g/kg ZBLE groups. Consequently, the persistence of the observed responses and the metabolomic dose–response pattern could not be determined. In addition, the chemical composition of botanical extracts may vary with raw-material source, maturity, and production batch. Future studies should conduct targeted quantification and functional validation of the principal differential lipids and evaluate the compositional consistency, long-term efficacy, and practical applicability of ZBLE under commercial production conditions.

## 5. Conclusions

Dietary supplementation with ZBLE improved laying rate and yolk color and modulated selected humoral immune-related parameters and antioxidant status in laying hens. The intestinal morphological responses differed among intestinal segments rather than showing a uniform improvement. Hepatic metabolomic comparison between the control and 6 g/kg ZBLE groups identified treatment-associated changes in lipid-related metabolism. Glycerophospholipid metabolism showed the highest pathway-impact value, and SM(d16:0/18:1) had the highest VIP value among the differential metabolites. Among the tested levels, 6 g/kg ZBLE produced the most favorable overall response. These findings support the potential use of *Z. armatum* DC. ‘Jiuyeqing’ leaves as a phytogenic feed resource for laying hens.

## Figures and Tables

**Table 1:** Composition and nutritional levels of the basal diet (air-dry basis, %).

Items	Basal Diet
Ingredients	
Corn	60.70
Soybean meal	22.00
Wheat bran	3.00
Soybean oil	3.00
Limestone	8.97
Dicalcium phosphate	1.40
L-Lysine·HCl	0.13
DL-Methionine	0.16
L-Threonine	0.02
Sodium chloride	0.25
Sodium bicarbonate	0.10
Choline chloride	0.10
Mineral premix ^1^	0.15
Vitamin premix ^2^	0.02
Total	100.00
Nutrient levels ^3^	
Metabolizable energy, MJ/kg	11.60
Crude protein	16.77
Calcium	3.86
Total phosphorus	0.58
Available phosphorus	0.32
Digestible amino acids	
Lysine	0.79
Methionine	0.37
Threonine	0.50
Tryptophan	0.15

^1^ Each kilogram of the diet contained the following mineral premix components: CuSO_4_·5H_2_O 8 mg, FeSO_4_·H_2_O 60 mg, MnSO_4_·H_2_O 60 mg, ZnSO_4_·H_2_O 80 mg, KI 0.35 mg, Na_2_SeO_3_ 0.30 mg. ^2^ Each kilogram of the diet contained the following vitamins supplied by the vitamin premix: retinol, 8000 IU; cholecalciferol, 1600 IU; tocopherol, 5 IU; thiamine, 0.8 mg; riboflavin, 2.5 mg; D-calcium pantothenate, 2.2 mg; pyridoxine, 3.0 mg; cyanocobalamin, 0.004 mg; menadione, 0.5 mg; biotin, 0.10 mg; and nicotinic acid, 20.0 mg. ^3^ Crude protein and total phosphorus were determined according to AOAC methods [20]. The remaining nutrient values were calculated according to the NRC (1994) [18] and the Chinese Feeding Standard of Chicken (NY/T 33-2004) [19].

**Table 2:** Effects of dietary ZBLE supplementation on laying performance in laying hens ^1^.

Items	*ZBLE* Supplementation, g/kg				SEM	*p*-Value		
	0	2	4	6		ANOVA	Linear	Quadratic
Weeks 1–4								
Laying rate, %	90.00	90.66	91.12	92.28	0.581	0.063	0.009	0.669
ADFI, g/hen/day	119.98	124.67	122.18	122.62	1.796	0.347	0.504	0.247
Average egg weight, g	59.64	60.00	60.22	61.62	1.085	0.598	0.216	0.636
Feed conversion ratio	2.02	2.08	2.04	1.99	0.039	0.432	0.469	0.168
Weeks 5–8								
Laying rate, %	89.46	89.18	89.60	91.30	0.816	0.269	0.114	0.237
ADFI, g/hen/day	120.37	123.27	121.47	121.17	1.595	0.628	0.931	0.325
Average egg weight, g	58.63	58.81	59.48	60.08	1.404	0.881	0.433	0.882
Feed conversion ratio	2.06	2.10	2.06	2.02	0.046	0.664	0.396	0.426
Weeks 1–8								
Laying rate, %	89.73 ^b^	89.92 ^ab^	90.36 ^ab^	91.79 ^a^	0.490	0.026	0.005	0.218
ADFI, g/hen/day	120.17	123.97	121.82	121.90	1.588	0.426	0.673	0.251
Average egg weight, g	59.14	59.41	59.85	60.85	1.163	0.743	0.294	0.757
Feed conversion ratio	2.04	2.09	2.05	2.01	0.039	0.501	0.412	0.246

^1^ ADFI, Average daily feed intake; ZBLE, *Zanthoxylum armatum* DC. ‘Jiuyeqing’ leaf extract; *n* = 8. ^a,b^ Mean values within a row with different superscript letters are significantly different (*p* < 0.05).

**Table 3:** Effects of dietary ZBLE supplementation on egg quality in laying hens ^1^.

Items	*ZBLE* Supplementation, g/kg				SEM	*p*-Value		
	0	2	4	6		ANOVA	Linear	Quadratic
Eggshell color								
L*	76.89	75.60	75.41	77.77	0.855	0.188	0.527	0.041
a*	5.38	4.92	4.76	5.36	0.339	0.469	0.895	0.125
b*	14.32	14.28	14.02	15.43	0.570	0.327	0.240	0.215
Egg length-to-width ratio	1.49	1.41	1.46	1.40	0.050	0.561	0.343	0.872
Eggshell strength, kg/cm^2^	4.31	4.59	4.30	4.77	0.209	0.336	0.255	0.655
Albumen height, mm	6.99	7.25	7.22	7.71	0.244	0.224	0.058	0.646
Yolk color	4.46 ^c^	4.92 ^bc^	5.63 ^ab^	5.76 ^a^	0.218	<0.001	<0.001	0.274
Haugh unit	83.16	85.08	83.38	87.73	1.479	0.131	0.080	0.419
Eggshell weight, g	5.85	6.04	5.89	6.03	0.138	0.703	0.558	0.840
Eggshell thickness, mm	0.34	0.35	0.34	0.34	0.007	0.759	0.933	0.808

^1^ ZBLE, *Zanthoxylum armatum* DC. ‘Jiuyeqing’ leaf extract; *n* = 8. ^a,b,c^ Mean values within a row with different superscript letters are significantly different (*p* < 0.05).

**Table 4:** Effects of dietary ZBLE supplementation on serum biochemistry and immunoglobulins in laying hens ^1^.

Items	*ZBLE* Supplementation, g/kg				SEM	*p*-Value		
	0	2	4	6		ANOVA	Linear	Quadratic
ALP, U/L	754.60	678.71	727.45	635.16	152.1	0.948	0.654	0.958
AST, U/L	200.09	180.11	215.41	197.44	13.30	0.334	0.649	0.940
Urea, mmol/L	0.62	0.62	0.67	0.61	0.054	0.846	0.968	0.594
ALT, U/L	3.02	2.98	2.46	3.12	0.622	0.880	0.943	0.580
ALB, g/L	16.99	21.38	21.29	20.61	1.284	0.070	0.071	0.058
CHO, mmol/L	2.66	3.25	2.05	1.87	0.504	0.222	0.123	0.447
TG, mmol/L	9.52	10.69	6.14	6.44	2.062	0.329	0.081	0.835
TP, g/L	54.88	62.50	56.31	55.00	4.954	0.665	0.795	0.375
IgA, mg/mL	58.07	62.63	67.62	72.81	5.007	0.209	0.036	0.951
IgM, mg/mL	276.75 ^b^	293.22 ^b^	343.11 ^ab^	380.56 ^a^	21.37	0.007	<0.001	0.627
IgG, mg/mL	102.22	121.07	109.72	124.28	8.624	0.264	0.054	0.806

^1^ ZBLE, *Zanthoxylum armatum* DC. ‘Jiuyeqing’ leaf extract; ALP, alkaline phosphatase; AST, aspartate aminotransferase; ALT, alanine aminotransferase; ALB, albumin; CHO, cholesterol; TG, triglyceride; TP, total protein; IgA, immunoglobulin A; IgM, immunoglobulin M; IgG, immunoglobulin G; *n* = 8. ^a,b^ Mean values within a row with different superscript letters are significantly different (*p* < 0.05).

**Table 5:** Effects of dietary ZBLE supplementation on organ weights and indices in laying hens ^1^.

Items	*ZBLE* Supplementation, g/kg				SEM	*p*-Value		
	0	2	4	6		ANOVA	Linear	Quadratic
Body weight, kg	1.67	1.58	1.76	1.53	0.068	0.096	0.418	0.309
Liver weight, g	39.53	38.71	41.28	37.29	2.153	0.625	0.671	0.468
Spleen weight, g	1.52	1.70	1.69	1.46	0.194	0.765	0.832	0.302
Ovarian weight, g	54.22	52.61	56.97	49.36	3.017	0.362	0.456	0.329
Oviduct weight, g	65.27	62.70	68.17	60.49	3.478	0.450	0.572	0.470
Liver index, %	2.37	2.48	2.35	2.45	0.143	0.900	0.846	0.983
Spleen index, %	0.09	0.11	0.10	0.10	0.012	0.832	0.974	0.509
Ovarian index, %	3.25	3.35	3.23	3.22	0.157	0.924	0.769	0.724
Oviduct index, %	3.90	4.01	3.90	3.99	0.234	0.979	0.886	0.957

^1^ ZBLE, *Zanthoxylum armatum* DC. ‘Jiuyeqing’ leaf extract; *n* = 8.

**Table 6:** Effects of dietary ZBLE supplementation on serum and hepatic antioxidant status in laying hens ^1^.

Items	*ZBLE* Supplementation, g/kg				SEM	*p*-Value		
	0	2	4	6		ANOVA	Linear	Quadratic
Serum								
T-AOC, U/mL	4.23 ^b^	6.18 ^a^	6.59 ^a^	6.25 ^a^	0.492	0.009	0.007	0.028
CAT, U/mL	10.49	9.45	11.54	10.74	1.103	0.615	0.571	0.911
MDA, nmol/mL	5.35 ^a^	4.32 ^ab^	4.11 ^ab^	2.99 ^b^	0.489	0.019	0.002	0.924
T-SOD, U/mL	107.56 ^b^	113.93 ^ab^	130.28 ^ab^	143.53 ^a^	6.899	0.004	<0.001	0.622
Liver								
T-AOC, U/mg prot	4.15 ^b^	5.22 ^ab^	5.33 ^ab^	6.32 ^a^	0.449	0.020	0.003	0.937
CAT, U/mg prot	8.48 ^b^	7.18 ^b^	6.91 ^b^	12.35 ^a^	0.827	<0.001	0.003	<0.001
MDA, nmol/mg prot	1.73 ^a^	1.35 ^ab^	0.96 ^b^	1.44 ^ab^	0.169	0.027	0.104	0.017
T-SOD, U/mg prot	709.06	770.14	734.63	827.32	56.75	0.496	0.219	0.783

^1^ ZBLE, *Zanthoxylum armatum* DC. ‘Jiuyeqing’ leaf extract; T-AOC, total antioxidant capacity; CAT, catalase activity; MDA, malondialdehyde content; T-SOD, total superoxide dismutase activity; *n* = 8. ^a,b^ Mean values within a row with different superscript letters are significantly different (*p* < 0.05).

**Table 7:** Effects of dietary ZBLE supplementation on intestinal morphology in laying hens ^1^.

Items	*ZBLE* Supplementation, g/kg				SEM	*p*-Value		
	0	2	4	6		ANOVA	Linear	Quadratic
Duodenum								
Villus height, µm	1050.2 ^b^	1177.6 ^b^	1577.8 ^a^	1601.4 ^a^	41.12	<0.001	<0.001	0.217
Crypt depth, µm	177.05	165.94	192.34	194.83	14.80	0.483	0.238	0.649
V/C	6.23	7.45	8.47	8.68	0.674	0.061	0.010	0.460
Jejunum								
Villus height, µm	1011.61	900.80	987.70	907.20	39.64	0.136	0.212	0.705
Crypt depth, µm	136.06 ^ab^	109.49 ^b^	131.70 ^ab^	155.03 ^a^	9.318	0.017	0.068	0.012
V/C	7.62 ^ab^	8.61 ^a^	7.70 ^ab^	5.94 ^b^	0.514	0.009	0.015	0.013
Ileum								
Villus height, µm	854.97	914.08	809.20	780.45	37.87	0.091	0.062	0.256
Crypt depth, µm	131.14	150.76	144.57	148.48	10.13	0.531	0.321	0.445
V/C	6.66	6.25	5.85	5.41	0.473	0.296	0.059	0.973

^1^ ZBLE, *Zanthoxylum armatum* DC. ‘Jiuyeqing’ leaf extract; V/C, Villus height/Crypt depth; *n* = 8. ^a,b^ Mean values within a row with different superscript letters are significantly different (*p* < 0.05).

## Data Availability

The original contributions presented in this study are included in the article. Further inquiries can be directed to the corresponding author(s).
